# Laparoendoscopic Single-Site Surgery for Management of Heterotopic Pregnancy: A Case Report and Review of Literature

**DOI:** 10.1155/2018/7232637

**Published:** 2018-07-04

**Authors:** Shadi Rezai, Richard A. Giovane, Heather Minton, Elise Bardawil, Yiming Zhang, Ninad M. Patil, Cassandra E. Henderson, Xiaoming Guan

**Affiliations:** ^1^Department of Obstetrics and Gynecology, Southern California Kaiser Permanente, Kern County, 1200 Discovery Drive, Bakersfield, CA 93309, USA; ^2^Division of Minimally Invasive Gynecologic Surgery, Department of Obstetrics and Gynecology, Baylor College of Medicine, 6651 Main Street, 10^th^ Floor, Houston, TX 77030, USA; ^3^University of Alabama, Department of Family Medicine, 801 Campus Drive, Tuscaloosa, AL 35487, USA; ^4^University of Birmingham, School of Medicine, 1720 2^nd^ Avenue, Birmingham, AL 35294, USA; ^5^Division of Reproductive Medicine, Jinan Central Hospital Group, 105 Jiefang Road, Jinan City, Shandong Province 250013, China; ^6^Department of Pathology & Immunology, Baylor College of Medicine, 6651 Main Street, 4^th^ Floor, Houston, TX 77030, USA; ^7^Maternal and Fetal Medicine, Department of Obstetrics and Gynecology, Lincoln Medical and Mental Health Center, 234 East 149^th^ Street, Bronx, NY 10451, USA

## Abstract

**Background:**

Heterotopic pregnancy occurs when two pregnancies occur simultaneously in the uterus and an ectopic location. Treatment includes removal of the ectopic pregnancy with preservation of the intrauterine pregnancy. Treatment is done laparoscopically with either a Laparoendoscopic Single-Site Surgery (LESS) or a multiport laparoscopic surgery.

**Case:**

We present a case of a first trimester heterotopic pregnancy in a 42-year-old gravida 5, para 0-1-3-1 female with previous history of left salpingectomy, who underwent laparoscopic right salpingectomy and lysis of adhesions (LOA) via Single-Incision Laparoscopic Surgery (SILS).

**Conclusion:**

Although LESS for benign OB/GYN cases is feasible, safe, and equally effective compared to the conventional laparoscopic techniques, studies have suggested no clinically relevant advantages in the frequency of perioperative complications between LESS and conventional methods. No data on the cost effectiveness of LESS versus conventional methods are available. LESS utilizes only one surgical incision which may lead to decreased pain and better cosmetic outcome when compared to multiport procedure. One significant undesirable aspect of LESS is the crowding of the surgical area as only one incision is made. Therefore, all instruments go through one port, which can lead to obstruction of the surgeon's vision and in some cases higher rate of procedure failure resulting in conversion to multiport procedure.

## 1. Background

Heterotopic pregnancy is defined as two simultaneous pregnancies that occur at different sites of implantation, most commonly uterine cavity and fallopian tube [1, 2]. The incidence of heterotopic pregnancy is 1 in 30,000. However, patients with fallopian tube disease have a greater risk of having heterotopic pregnancy [3]. Presentation is similar to an ectopic pregnancy, including flank pain, vaginal bleeding, and, in severe cases, hemodynamic instability [4]. Diagnosis is often made via transvaginal ultrasound [4]. Treatment includes removal of the ectopic pregnancy with preservation of the intrauterine pregnancy [3, 4]. Frequent management is to remove the ectopic pregnancy via a laparoscopic approach using a single incision or multiple port approach [5, 6]. Employing the single-site laparoscopic procedure can be a more favorable approach due to its simplicity [7].

Laparoendoscopic Single-Site Surgery (LESS) is a form of surgery in which a single incision is made, usually at the umbilicus [8–10]. Although this technique has been referred to by different names, LESS is now the accepted name of this procedure by consensus [11]. LESS has been used for different procedures such as cholecystectomy, appendectomy, and ectopic pregnancy [12]. LESS is generally a more favored approach than laparotomy due to the patient having less postoperative pain, better cosmetic results, and shorter hospital stay [13]. Furthermore, as LESS uses a single port for surgery, there is less of a risk for infection and blood loss [14]. There are, however, disadvantages to this technique such as only having one port for placement of the camera and instruments, which hinders depth perception and decreases the field of view [15].

## 2. Presentation of the Case

Our patient is a 42-year-old, obese, gravida 5, para 0-1-3-1 woman, who was referred to our clinic for laparoscopic management of heterotopic pregnancy. The patient had a history of adverse perinatal outcome and poor obstetric history with one preterm classical cesarean delivery at 25 6/7 weeks in 2014. The patient was diagnosed with female infertility of unspecified origin in 2012 that was being managed by the Reproductive Endocrinology and Infertility (REI) service with the use of stored eggs and in vitro fertilization (IVF). Of note, the patient was also being managed by the Maternal-Fetal Medicine (MFM) service for a history of first trimester recurrent pregnancy losses (RPL) at 4 weeks, 6 weeks, and 11 weeks of gestational age (GA) that included a twin gestation. The RPL work-up revealed a clotting disorder (MTHFR C677T single copy) and hypothyroidism. Relevant surgical history for the patient was a left salpingectomy in 2000.

The patient was planning to attempt embryo transfer in July 2017 using stored eggs from previous in vitro fertilization (IVF) cycle.

**Figure 1:**
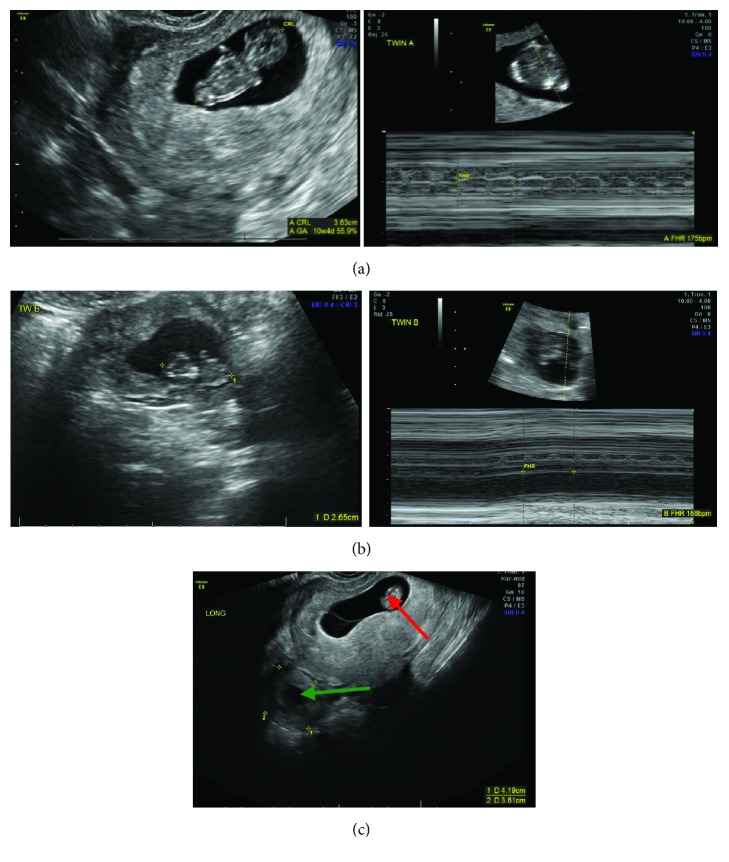
Official ultrasound showing positive FHR for Twin A, intrauterine pregnancy (IUP) (**a**); for Twin B, right tubal ectopic pregnancy (EP) (**b**); and presence of both** Twin A [intrauterine pregnancy (IUP)] (red arrow)** and** Twin B [ectopic pregnancy (EP)] (green arrow)** (**c**).

Obstetrics ultrasound** [Figures 1(a), 1(b), and 1(c)] **revealed one intrauterine pregnancy (IUP) (Twin A) at 10 4/7 weeks with fetal heart rate (FHR) of 175 BPM with Crown-Rump Length (CRL) of 36 mm corresponding to the 56^th^ percentile** [Figure 1(a)]**, as well as one right ectopic tubal pregnancy (Twin B) at 9 3/7 weeks with CRL of 26.5 mm corresponding to less than 5^th^ percentile with FHR of 188 BPM** [Figure 1(b)].**

**Figure 2:**
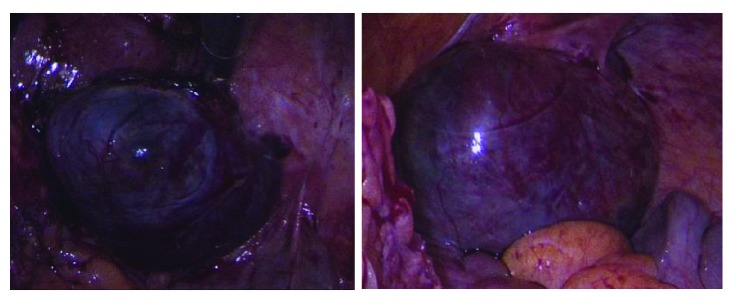
Intraoperative laparoscopic images of heterotopic pregnancy showing right tubal heterotopic pregnancy.

The patient underwent laparoscopic right salpingectomy and lysis of adhesions (LOA) via Single-Incision Laparoscopic Surgery (SILS) with estimated blood loss of 10 ml** [Figure 2]**. SILS was done via inserting a single-site laparoscopy device at a 15 mm umbilical port [16]. The right fallopian tube with ectopic pregnancy was identified and LOA performed using the ENSEAL® articulating tissue sealer and the Endo Shears. Slight bleeding from the tube was controlled using the bipolar device. The fallopian tube was transected near the cornua.

Following surgery, the specimen was opened with identification of the presence of fetus and placental tissue. Transvaginal ultrasound (TVUS) following surgery confirmed the presence of a detectable fetal heart beat in the intrauterine pregnancy at 165 BPM and the presence of positive fetal movement. The patient did not require anti-D immune globulin as the patient had an O Rh positive blood type. The patient had an uncomplicated recovery course and was discharged on postoperative day 1.

The pathology specimen was examined with disrupted right fallopian tube with tubally implanted placenta and embryo that were consistent with a diagnosis of ectopic pregnancy** [Table 1].**

Regarding the intrauterine pregnancy (Twin A), the patient underwent repeat cesarean delivery at 36 5/7 weeks of gestation due to previous classical cesarean delivery and microscopic placenta accreta. Estimated blood loss (EBL) was 1000 ml. The patient gave birth to a healthy baby with weight of 2820 grams and APGAR scores of 8 and 9 at 1 and 5 minutes, respectively. The postoperative course was uncomplicated after her cesarean delivery, with hospital discharge occurring on postoperative day 3.

## 3. Discussions

Heterotopic pregnancy is two simultaneous pregnancies in which one occurs in the uterus and the other occurs in an ectopic location [2]. Patients who have tubal disease and increased levels of estrogen and progesterone have an increased risk of developing heterotopic pregnancy [17]. Patient presentation may be variable. They generally present with abdominal or flank pain, vaginal bleeding, or, in advanced cases, shock. Differentiating heterotopic pregnancy from ectopic pregnancy is of critical importance as management will differ. Ultrasound is used to differentiate ectopic from heterotopic pregnancy, due to the presence of an additional gestational sac in the uterine cavity [18, 19]. The use of measured beta-human chorionic gonadotrophin has little utility in diagnosing a heterotopic pregnancy as levels will reflect that of the intrauterine pregnancy [11]. Management is surgical with the goal of removing the ectopic pregnancy [6, 20, 21]. The choice of laparotomy versus a laparoscopic procedure is dependent on the patient's hemodynamic stability. Furthermore, a LESS approach versus multiport approach is dependent on certain factors as outlined below.

LESS was first introduced for ectopic pregnancy treatment by Ghezzi et al. [17]. Although the management can include medical management through the use of methotrexate, situations arise when surgical management is the only option such as when the patient is hemodynamically unstable or fetal heart beats are detected. The decision to use LESS versus multiple ports depends on different factors pertaining to the patient. One absolute contraindication for LESS is if the patient has an abdominal mesh from a prior umbilical hernia repair [22]. Theoretically, LESS is a simpler procedure as only one incision is made; however, a major drawback to this surgery as that only one port is placed so there is difficulty maneuvering instruments in the abdomen as well as gauging depth perception [23, 24]. Comparing LESS to multiport surgeries is important when determining which method to use. Regarding length of time to completion of surgery, LESS has been shown to require less time to complete than multiport surgery in ectopic pregnancy [15, 17]. Regarding adverse events, LESS had comparable events to multiport surgeries [25, 26]. However, it is suggested that LESS has an increased rate of umbilical hernia when compared to multiport surgery [18, 26]. Regarding hospital stay and postoperative pain, patients undergoing LESS have shorter hospital stay and have less pain [15]. Furthermore, patients who underwent LESS had more favorable cosmetic results [15].

## 4. Conclusion

Heterotopic pregnancy is a rare form of pregnancy in which two simultaneously pregnancies occur, one intrauterine and one in an ectopic location. Treatment is directed at removing the ectopic pregnancy while trying to preserve the intrauterine pregnancy. Removal of the ectopic pregnancy is done laparoscopically if the patient is hemodynamically stable. LESS has become a viable option, as only it requires only one incision which leads to fewer surgical complications such as bleeding, infection, postoperative pain [27, 28], and hospital stay.

Although LESS for benign OB/GYN cases is feasible, safe, and equally effective compared to the conventional laparoscopic techniques [16, 29], studies have suggested no clinically relevant advantages in the frequency of perioperative complications between LESS and conventional methods [29, 30]. No data on the cost effectiveness of LESS versus conventional methods are available [29].

LESS utilizes only one surgical incision which may lead to decreased pain and better cosmetic outcome when compared to multiport procedure.

Disadvantages associated with LESS include possible difficulty in maneuvering instruments in one port due to loss of triangulation as well as obstruction of view and in some cases higher rate of procedure failure resulting in conversion to multiport procedure [29]. A case by case approach must be adopted when deciding to do LESS in a patient with heterotopic pregnancy. As far as future pregnancy for patient with history of heterotopic pregnancy, preconceptional counseling and planned pregnancy with early ultrasound imaging are recommended to ensure proper uterine implantation.

## Figures and Tables

**Table 1:** Pathology macroscopic and microscopic images of right fallopian tube with implanted placenta and fetus, consistent with ectopic pregnancy.

**Slide Number**	**Slide**	**Explanations**
**3A.**	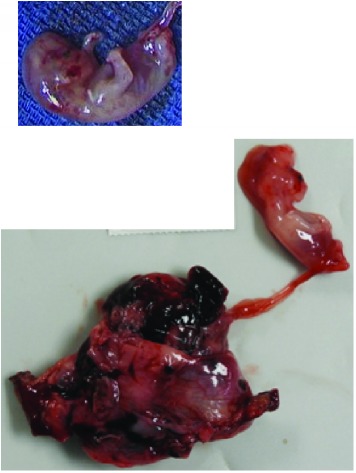	Right fallopian tube with implanted placenta, (bottom left) attached by umbilical cord to fetus.

**3B**	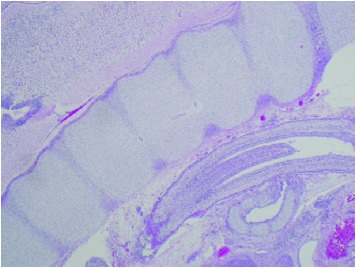	Fetal skeletal elements (Microscopy: hematoxylin and eosine stain).

**3C.**	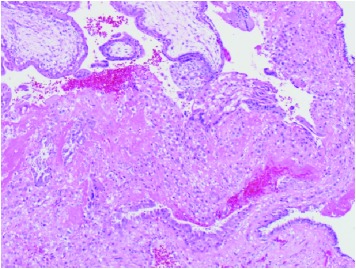	Placental chorionic villi (top left) with ectopic implantation (center) in tubal mucosa (bottom). (Microscopy: hematoxylin and eosine stain)
